# Optical properties of NIR photoluminescent PbS nanocrystal-based three-dimensional networks†

**DOI:** 10.1039/d3na00404j

**Published:** 2023-08-14

**Authors:** Denis Pluta, Henning Kuper, Rebecca T. Graf, Christoph Wesemann, Pascal Rusch, Joerg August Becker, Nadja C. Bigall

**Affiliations:** a Institute of Physical Chemistry and Electrochemistry, Leibniz University Hannover Callinstraße 3A 30167 Hannover Germany nadja.bigall@pci.uni-hannover.de; b Laboratory of Nano and Quantum Engineering, Leibniz University Hannover Schneiderberg 39 30167 Hannover Germany

## Abstract

The assembly of nanocrystals (NCs) into three-dimensional network structures is a recently established strategy to produce macroscopic materials with nanoscopic properties. These networks can be formed by the controlled destabilization of NC colloids and subsequent supercritical drying to obtain NC-based aerogels. Even though this strategy has been used for many different semiconductor NCs, the emission of NC-based aerogels is limited to the ultraviolet and visible and no near-infrared (NIR) emitting NC-based aerogels have been investigated in literature until now. In the present work we have optimized a gelation route of NIR emitting PbS and PbS/CdS quantum dots (QDs) by means of a recently established gel formation method using trivalent ions to induce the network formation. Thereby, depending on the surface ligands and QDs used the resulting network structure is different. We propose, that the ligand affinity to the nanocrystal surface plays an essential role during network formation, which is supported by theoretical calculations. The optical properties were investigated with a focus on their steady-state and time resolved photoluminescence (PL). Unlike in PbS/CdS aerogels, the absorption of PbS aerogels and their PL shift strongly. For all aerogels the PL lifetimes are reduced in comparison to those of the building blocks with this reduction being especially pronounced in the PbS aerogels.

## Abstract 2

NIR photoluminescent PbS nanocrystal-based aerogels are synthesized and characterized for the first time. The influence of surface facets and surface ligands on the resulting network structure is investigated and related to their optical properties.

## Graphical abstract

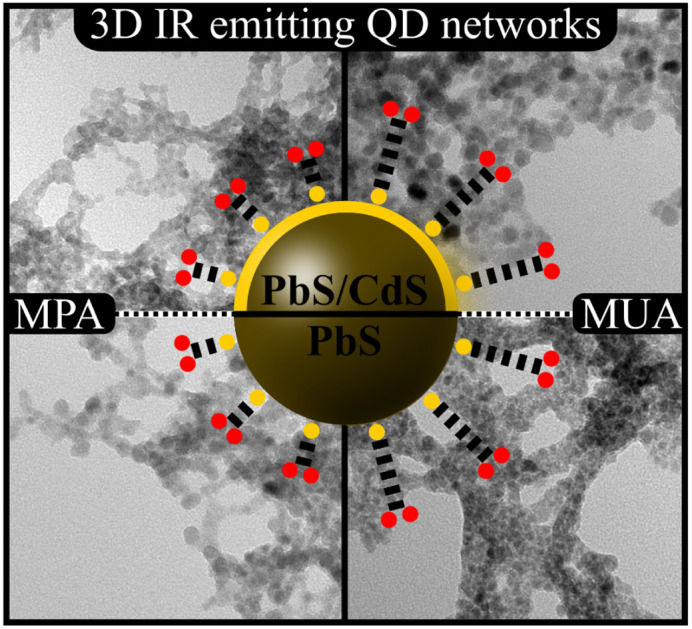

## Introduction

Semiconductor nanocrystals (NCs) have been studied intensely in the past decades due to their interesting properties originating from nanoscopic effects and immense synthetic control. This led to the ability to synthesize core/shell, alloyed, and 2D NCs to only name a few.^1–3^ Especially for nanocrystal-based materials with optical properties in the UV-vis range of the electromagnetic spectrum huge amounts of research has been conducted, whereas in comparison the infrared (IR) active materials are lacking far behind. While IR active materials are of high interest in multiple applications such as optoelectronic devices,^4,5^ photovoltaics,^6,7^ light-emitting diodes,^8^ telecommunications and many more, the syntheses and characterization methods for these materials are challenging.^9–13^ In photovoltaics for example, narrow band gap QDs are interesting because effects like *e.g.* multiple exciton generation show tremendous potential for high efficiency solar cells and photoelectrochemical hydrogen evolution.^6,13^ For telecommunication applications with, the telecommunication band between 1200 and 1700 nm, infrared QDs are suitable candidates spanning the whole spectral region. The same is true for deep tissue imaging with high-transparency spectral bands at 840 nm, 1110 nm, 1320 nm and 1680 nm.^9^

In addition to the challenges associated with NIR materials specifically, NCs are in general inherently difficult to implement into applications as colloids. This has been tackled, by using assemblies of NCs, of which NC-based aerogels are one of the most promising candidates. NC aerogels are three-dimensional network structures, that can be obtained by controlled destabilization of NCs and subsequent supercritical drying.^14–19^ They are not only macroscopic materials with retained nanoscopic properties, but in addition show new properties due to the interaction of the assembled NCs.^14–16,20–22^ Even though previous works made efforts to synthesize aerogels from infrared NCs, such as PbS, PbSe and PbTe, their properties and especially their photoluminescence (PL) have never been investigated in depth.^17,19,23–27^ So far, only in one single case, the NIR-emission of an acetogel has been reported, which was synthesized by cation exchange. However, the optical properties were only investigated superficially, since the investigations focused onto the structural properties and the cation exchange itself.^28^ Therefore, the direct synthesis of aerogels from infrared-emitting NCs and the characterization and optimization of their optical properties and especially their PL will bring these materials closer to application, *e.g.* the incorporation into QD solar cells to improve the transport of charge carriers within the QD layer in comparison to QD layers consisting of individual QDs. In addition, these studies will give an insight into the electronic structure and could lead to the understanding of observed nanoscopic effects.

**Fig. 1:**
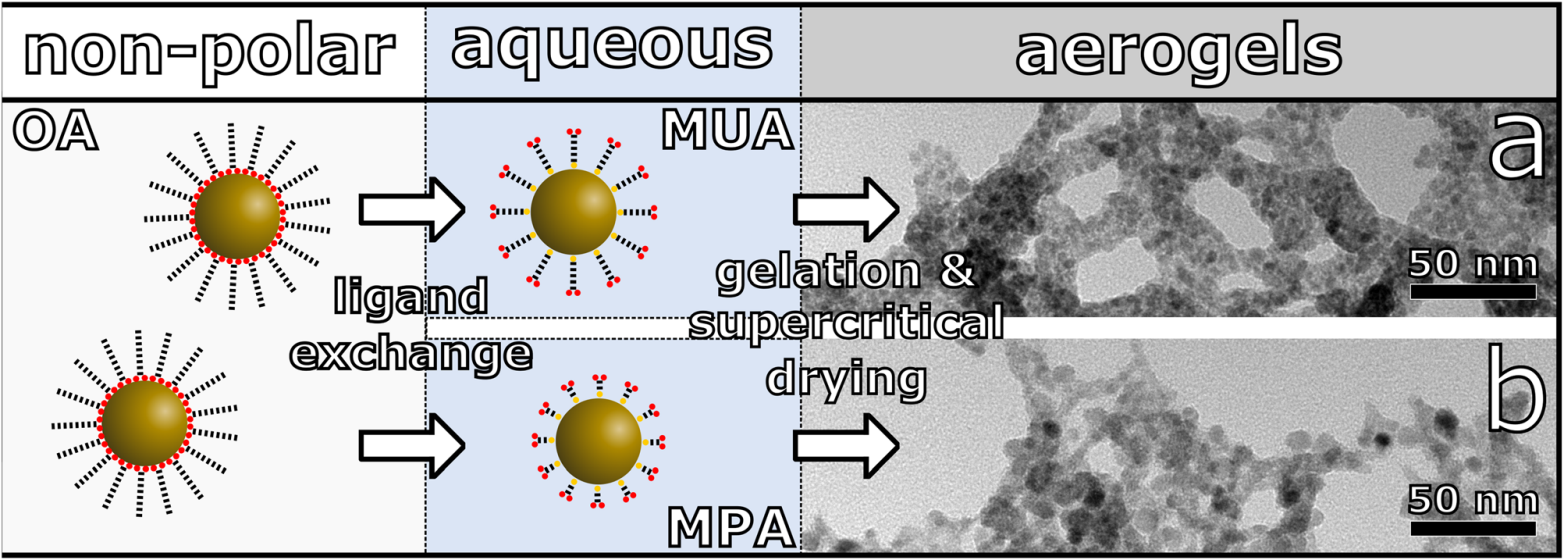
Schematic synthesis route from PbS QDs stabilized with oleic acid (OA) ligands to PbS nanocrystal based aerogels. The QDs are phase transferred to the aqueous phase *via* ligand exchange with 11-mercaptoundecanoic acid (MUA) or 3-mercaptopropionic acid (MPA), leading to different gel structures. TEM image of a PbS aerogel, assembled from (a) MUA and (b) MPA stabilized PbS QDs.

One of the most investigated classes of NIR-emitting NCs are Pb-chalcogenide NCs. This is due to comparably high synthetic control and outstanding optical properties including high photoluminescence quantum yields (PLQY).^10,12,29–31^ For that reason, we chose PbS NCs and core/shell PbS/CdS NCs as our starting materials for the synthesis of the three-dimensional assemblies. These NCs were synthesized according to recently reported literature procedures.^12,29^ The as-synthesized NCs were then transferred to the aqueous phase by ligand exchange with mercaptocarboxylic acid followed by controlled destabilization by addition of trivalent ions leading to the network formation, which is shown schematically in Fig. 1.^32,33^ The obtained hydrogels are washed multiple times with water, then the solvent is exchanged to acetone for supercritical drying with CO_2_ in order to receive aerogels. We show, that depending on the surface ligands and particle surface, different gel networks are synthesized, which are investigated by transmission electron microscopy (TEM) and scanning electron microscopy (SEM). Furthermore, this also has an influence on the optical properties and charge carrier dynamics, which are investigated with steady-state absorption and emission spectroscopy and time-resolved PL measurements.

## Results and discussion

### Particle synthesis, phase transfer and gelation optimization

The PbS QDs used for the fabrication of PbS aerogels have been synthesized following a procedure from Zhang *et al.*,^12^ which is a modified method from Hines and Scholes.^29^ We adapted the synthesis parameters to yield QDs with photoluminescence (PL) at 1215 nm (1.02 eV) with a PLQY of 23%. From TEM images a mean particle size of 3.8 ± 0.4 nm was derived (see Fig. S1a in the ESI†).

Furthermore, core/shell PbS/CdS QDs have been synthesized for the fabrication of core/shell PbS/CdS aerogels following a procedure from Zhao *et al.*,^31^ in which the outer layers of the synthesized PbS QDs are cation exchanged to CdS. The resulting QDs have a size of 3.9 ± 0.6 nm (Fig. S3a, ESI†) and are therefore similar in size to the starting PbS QDs (the measured difference is within the error of measurement of the particle size with TEM). On the other hand, their optical properties undergo a hypsochromic shift. This is the case because the PbS core, which is responsible for the low energy optical properties, is effectively shrunk during the cation exchange close to the particles surface. Since the thickness of the CdS shell is not visible in TEM due to low contrast, the size-dependent optical properties of PbS QDs investigated by Moreels *et al.*^34^ and their sizing curve can be used to estimate the PbS core size.^35^ The difference between the calculated sizes for the PbS and PbS/CdS QDs is 0.8 nm, which implies a CdS shell thickness of 0.4 nm, equivalent to roughly 1 layer of CdS of 0.35 nm.^36^ The core–shell structure, even for thin-shell PbS/CdS QDs was observed in TEM from Zhao *et al.*^31^ The PL of the PbS/CdS QDs is centered at 1087 nm (1.140 eV), corresponding to a shift of 128 nm compared to the pristine PbS QDs. The PLQY was measured to be 19%, which is lower in comparison to the core only PbS QDs. This decrease has also been observed for the growth of a CdS shell on PbS cores using colloidal atomic layer deposition (cALD), representing the extreme case of growing a CdS shell at lower temperatures.^37,38^ In our case the cation exchange takes place at 100 °C, which is still cold in comparison to hot injection or SILAR methods, where CdS is grown at up to 300 °C.^39^

The PbS and PbS/CdS QDs were then transferred from organic to aqueous phase *via* ligand exchange with 11-mercaptoundecanoic acid (MUA) and 3-mercaptopropionic acid (MPA) following a modified procedure from Kodanek *et al.* with optimized reaction parameters for the used PbS and core/shell PbS/CdS QDs.^32^ The TEM images for the phase transferred samples can be found in Fig. S1 and S3.† The size distribution of the PbS QDs before and after ligand exchange can be found in Fig. S2 in the ESI.† The mean diameter of the PbS MUA QDs and PbS MPA QDs is 3.3 ± 0.5 nm and 5.7 ± 1.7 nm, respectively. Thus, 0.5 nm smaller and 1.9 nm bigger than the as-synthesized PbS oleic acid (OA) QDs. This, at first glance, is contradictory to the results from absorbance measurements, which can be seen in Fig. S5 in the ESI.† The measured decrease in size for the PbS MUA QDs of 0.5 nm would lead to a hypsochromic shift of roughly 100 nm. Instead, only a small hypsochromic shift of the first excitation maximum of 19 nm from PbS OA to PbS MUA and 38 nm from PbS OA to PbS MPA can be observed in the absorbance spectra. This leads to the conclusion, that the measured decreased size for PbS MUA QDs is most likely due to measurement inaccuracy because of low contrast in the TEM images. For the PbS MPA QDs, the drastic measured size increase should analogously lead to a bathochromic shift of the absorbance. As this is not measured either, the increased size in the TEM should not be caused by the ligand exchange but different measurement factors. In Fig. S1c and d in the ESI† two TEM images of the PbS MPA QDs, which have been recorded in quick succession at the same position, are shown. In the highlighted areas, the QDs are coalescing during the measurement, which leads to the increase in measured particle size. Thus, the measured difference in size can most likely be attributed to fusion of particles during the TEM measurements itself and not during the ligand exchange, being in good agreement with results from the absorbance measurements. The fusion during the TEM measurements cannot be observed for the PbS OA or PbS MUA sample, likely due to the higher steric demands of these longer ligands.

The PL of the PbS QDs is significantly quenched after ligand exchange. The emission spectra can be found in Fig. S6 in the ESI.† For the PbS MPA QDs no emission was measured, while the PbS MUA QDs emission has a small hypsochromic shift which is in line with the shift observed in the absorbance measurements, again hinting at the stronger passivation by the longer chain ligands. The decrease in PL intensity is in good accordance to the observed decrease of PLQY in literature.^32,40^ For the core/shell PbS/CdS QDs the influence of the ligand exchange onto the optical properties is less pronounced. The absorbance and emission spectra can be found in Fig. S7 and S8 in the ESI.† A small hypsochromic shift of the absorbance can be measured, similar to the previously discussed PbS QDs. The negligible shift of the emission can be explained by the passivation of the PbS core with the wider bandgap CdS shell. This is supported by the high PL intensity compared to the core only PbS MPA and MUA QDs. The change of the solvent is accompanied by an increase of the dielectric constant of the QDs environment, which could also be responsible for the observed hypsochromic shift, as previously reported in literature.^41^ TEM images of the core/shell PbS/CdS OA, MUA and MPA QDs are shown in Fig. S3 in the ESI.† The changes in size are negligible, as shown in the measured size distributions in Fig. S4.† The measured size for the core/shell PbS/CdS OA, MUA and MPA QDs are 3.9 ± 0.6 nm, 3.6 ± 0.4 nm and 4.0 ± 0.4 nm, respectively. Ligand exchange or removal can lead to the removal of surface cations, which is especially relevant for QDs with thin shells, since the shell integrity could be compromised. It was shown, that the band gap of cation terminated metal chalcogenide QDs, such as PbS and PbS/CdS core–shell QDs is only marginally influenced by the removal of the mentioned surface cations.^37,42^ Since the thickness of the CdS shell has been determined by the difference in band gap energy between PbS and PbS/CdS QDs, the type of surface cation termination has no influence on the calculated shell thickness. The removal of surface Cd cations from PbS/CdS core–shell QDs due to ligand exchange or removal would therefore not compromise the shell integrity, since the electronically relevant part of the shell would still be in place. In summary, the synthesized QD building blocks show optical properties in the NIR, which are retained after phase transfer with MUA and MPA. Except for the PbS MPA QDs, PL was measured and exhibit only small hypsochromic shifts compared to the respective OA covered NCs.

The gelation of the phase transferred NCs was carried out following a procedure from Zámbó *et al.*^33^ using YCl_3_ and YbCl_3_ as the gelation agent. Hereby, YCl_3_ yielded solvogels with less shrinkage in comparison to YbCl_3_ and additionally, the measured PL of the resulting aerogels are brighter for YCl_3_ samples. To optimize the gelation different concentrations of YCl_3_ and YbCl_3_ have been used and the optimal concentration (meaning the least shrinkage and at the same time complete gelation) found was 75 mM corresponding to an atomic-Pb–Y-ratio of roughly 3.4 : 1. In Fig. S12 in the ESI† the resulting washed PbS hydrogels gellated with YCl_3_ and YbCl_3_ concentrations between 25 and 200 mM can be seen. For the samples, treated with 25 and 50 mM solutions the gelation is not complete, while for the other concentrations complete gelation can be observed. For these samples gelation time increases with decreasing YCl_3_ and YbCl_3_ concentration. For the samples synthesized with 200 mM solutions gelation was observed instantly, with 100 mM complete gelation was visible after 17 h and with 75 mM gelation took approximately 40 h. Furthermore, the volume of the 200 mM gels is significantly smaller compared to the 75 and 100 mM samples. This is most likely due to the fast kinetics of gelation, as quickly formed fragile networks might be destroyed early in the process by a vortexing homogenization step after addition of the trivalent ions. This can also have a minor influence onto the 100 mM sample, explaining the small difference in volume between the 100 and 75 mM gels. Regardless of concentration, the gels synthesized with YCl_3_ shrunk less during gelation compared to YbCl_3_ gellated ones.

For the core/shell PbS/CdS samples, gelation was not complete after 40 h with a 75 mM solution of YCl_3_. To achieve complete gelation for these samples, additional YCl_3_ solution was added after 17 h. After another 24 h complete gelation was observed. This could be explained with the superior stability of the core/shell PbS/CdS MUA and MPA QDs in comparison to their PbS counterparts. The differences between the QD building blocks used during gelation lead to different network structures, which are discussed in the following paragraph.

### Network structure

The aerogels form voluminous, porous macroscopic solids, which can be seen in the SEM images in Fig. S11 in the ESI.†

**Fig. 2:**
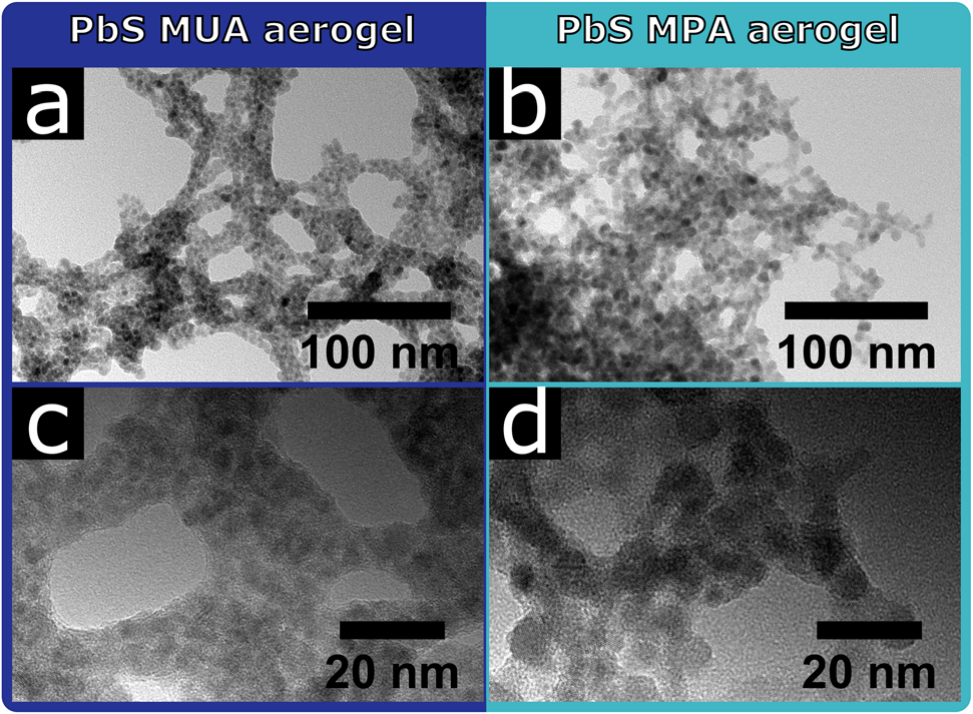
The network structure of the PbS aerogels depends on the surface ligands (MUA or MPA) used during the phase transfer step. TEM images of a PbS aerogel synthesized from QDs and with YCl_3_, stabilized with (a and c) MUA, and (b and d) MPA.

For the PbS QDs the influence of the surface ligands on the resulting network structure can clearly be seen in the TEM images shown in Fig. 2. The PbS MUA aerogel consists of a network of roughly 10–15 nm thick branches, that are interconnected and form bigger pores compared to the PbS MPA aerogel. Non-crystalline residue can be seen between and around the PbS MUA QDs. This suggests organic residues between the particles, in which the PbS QDs are embedded as small aggregates. This is not the case for the PbS MPA aerogel, where non-crystalline residue cannot be seen and the network structure is denser, crystal contacts between QDs are visible and in some cases multiple particles are coalesced.

Since the synthesis parameters were kept the same for the PbS MUA and MPA aerogels, except for the ligands used during the phase transfer, the observed difference in structure is likely caused by the present surface ligands. The significant difference between MUA and MPA is their carbon chain length (C11 and C3 chain, respectively), and upon addition of YCl_3_ the ligands are coordinated by Y^3+^ and particles are interconnected *via* ligand–Y^3+^–ligand complexes, which has been shown by XPS measurements previously.^33^ Since the distance between particles is limited by the ligand length, MPA stabilized particles are significantly closer to each other, which makes a particle contact more likely, compared to the MUA stabilized sample. Thus, the ligand length might be a probable explanation for the observed differences in the PbS network structures.

**Fig. 3:**
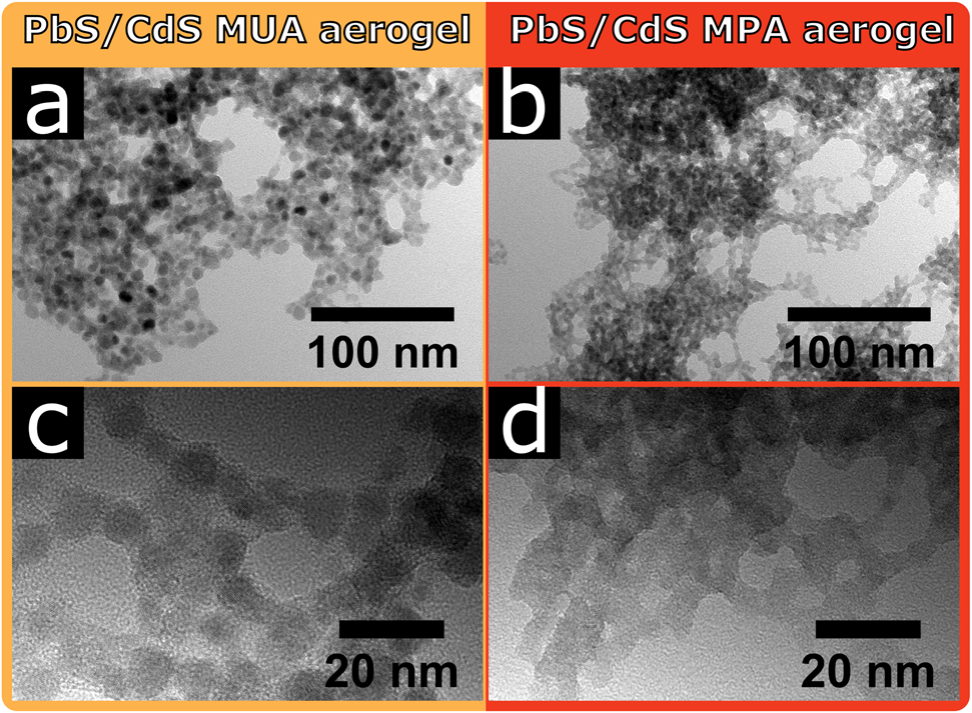
The PbS/CdS aerogels show, similarly to the PbS aerogels, a dependency of the surface ligands onto the obtained network structure. In addition, the particle type is also relevant. TEM images of a PbS/CdS aerogel synthesized from QDs, stabilized with (a and c) MUA and (b and d) MPA.

In Fig. 3 the TEM images of the core/shell PbS/CdS MUA and MPA aerogels are shown. For the core/shell PbS/CdS MUA aerogel individual particles are visible and appear to be in crystal contact. However, at higher magnifications some areas are visible, where non-crystalline material is connecting the PbS/CdS MUA particles as could be seen for the PbS MUA aerogel. The core/shell PbS/CdS MPA aerogel on the other hand is comparably unique, since the building blocks are so strongly coalesced, that individual particles are barely visible. For both core/shell PbS/CdS aerogel samples the ligand removal during the gelation seems to be significantly more efficient compared to the PbS aerogels, manifesting in the resulting gel network structures. Nevertheless, the above observed tendency of MPA facilitating more pronounced crystal contact and coalesced particles in comparison to MUA for the PbS aerogels is also observed with the core/shell PbS/CdS samples. At the same time the fact, that the network structures differ when using PbS or core/shell PbS/CdS QDs proves that not only the ligands are relevant during the gelation, but also their interaction with the particle surface.

### Ligand surface interactions

Ligand removal is known to play a vital role in nanoparticle gelation, revealing reactive surfaces for condensation of NCs to networks. For ligand destructive methods, such as the gelation with H_2_O_2_, the thiol groups of the surface ligands are oxidized, which leads to the dissociation of the ligands from the particle surface.^19^ For non-destructive gelation agents such as complexing cations as applied in this work, the successful ligand removal is dependent on the bond strength between ligands and particle and therefore dependent on the particle surface. Here, the nanoparticle surface is altered during the cation exchange procedure and instead of rock salt PbS with (111) and (100) facets the surface is dominated by zinc blende CdS(111) and (100) facets in the PbS/CdS core/shell NCs.^31,43,44^ These different surfaces and thus different ligand–surface interactions can be a possible explanation for the discussed differences in this work, namely the PbS networks containing significant amounts of ligands, while the PbS/CdS networks do not and will be discussed in the following.

It is reported in literature that experimentally synthesized PbS nanocrystals with sizes less than 2.7 nm are completely enclosed by lead terminated (111) facets while larger cuboctahedral PbS nanocrystals with size up to 7.5 nm have truncated (100) facets in addition to (111) facets.^43^ Therefore, the surfaces of the PbS particles with a diameter of 3.8 nm synthesized in this work will possess mainly (111) facets with a small fraction of (100) facets. Zinc blende CdSe nanocrystals with a size of less than 4 nm are known to exhibit exclusively CdSe(100) facets.^44^ Since the lattice constant of zinc blende CdSe is only about 4.5% larger than the lattice constant of zinc blende CdS and the PbS/CdS QDs synthesized in this work have a size of 3.7 nm, it can be assumed that the PbS/CdS QDs are mainly, if not exclusively terminated with CdS(100) facets.^45–47^

**Fig. 4:**
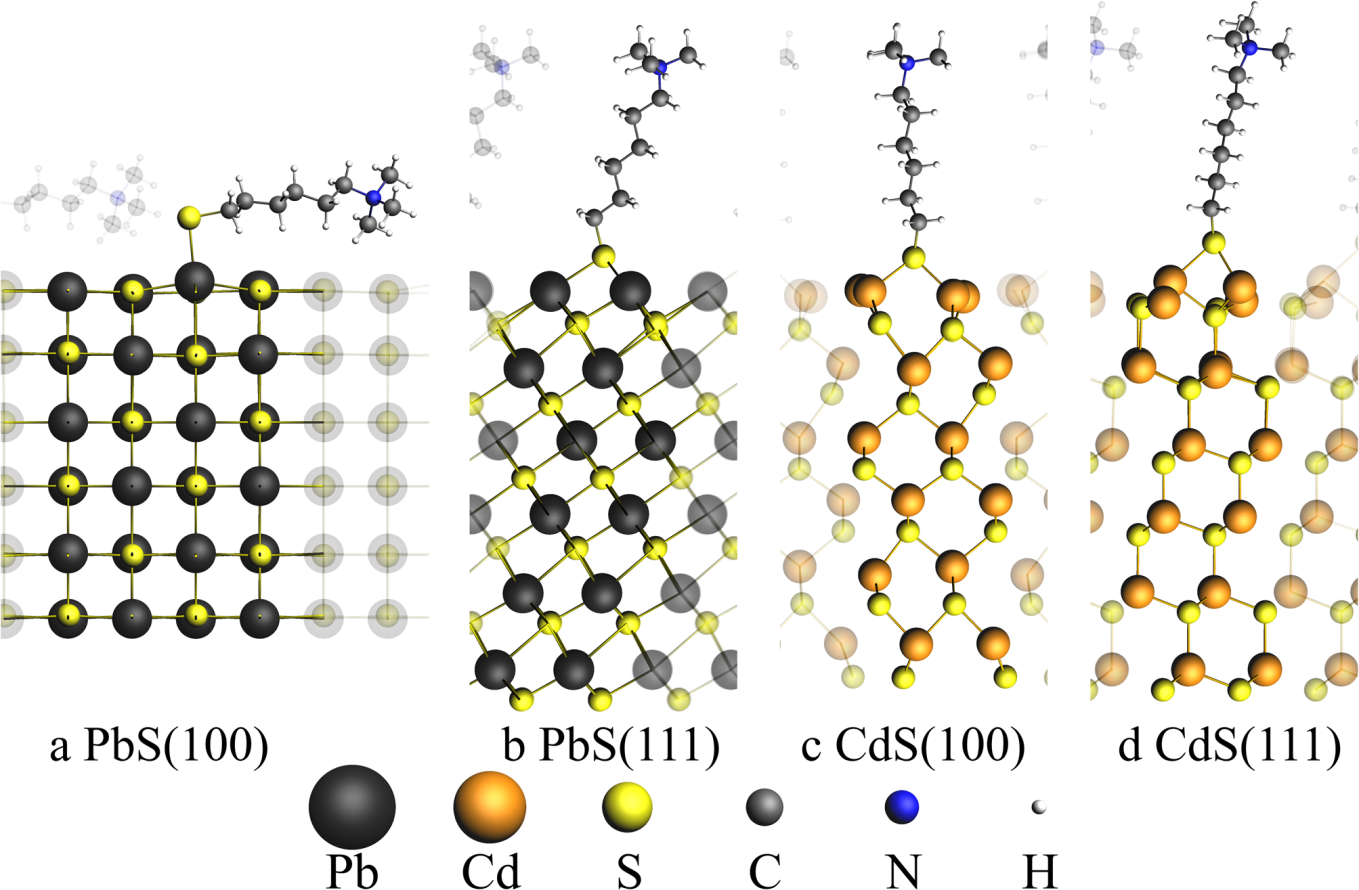
Illustration of four surface structures used to calculate absorption energies. (a) and (b) show the PbS rock salt surfaces with (100) and (111) surfaces respectively, whereas (c) and (d) show the CdS zinc blende (100) and (111) surfaces respectively. Neighboring simulation cells were show with transparent atoms. The thiol ligand is composed of a sulfur atom, a *n*-hexylchain and a trimethylamine group (S–(CH_2_)_6_–N–(CH_3_)_3_). For (a) an adsorption site of the ligand sulfur atom on top of the lead atom is found. For all other three structures bridged adsorption sites were found where the sulfur atom is bound to two Pb/Cd atoms.

It is therefore interesting to investigate the bond strength between ligand and surface within a theoretical model for PbS in the rock salt crystal structure and for CdS in the zinc blend structure and its reactions with a model thiol ligand molecule specified in the ESI.† This aims to study the reactionPbS–thiol-ligand → PbS + thiol-ligandin order to determine the adsorption energy Δ*E*_ads_. Illustrations of the model structures can be seen in Fig. 4 and details about the calculation are provided in the ESI.†

A total of four surfaces were generated as slabs with the two surface types (100) and (111) for PbS and CdS, respectively. Each surface was optimized together with the model thiol ligand using *ab initio* methods with BAND implemented in the SCM suite on PBE and relativistic double zeta level of theory.^46–50^

Table 1 shows the calculated absorption energies for the four surfaces and the type of adsorption site which was found for the thiol ligand at the surface.

**Table 1:** Adsorption energies and the type of adsorption site are shown for the four surface types illustrated in Fig. 4

	PbS(100)	PbS(111)	CdS(100)	CdS(111)
Δ*E*_ads_/kJ mol^−1^	−339.04	−297.34	−245.03	−196.15
Adsorption site	On top	Bridge	Bridge	Bridge

Unlike the other discussed facets, only the PbS(100) facet does exhibit a mixed cation–anion terminated surface, while all other facets are purely terminated by cations. This leads to an on top adsorption site only found for PbS(100) with an adsorption energy of −339.04 kJ mol^−1^. Adsorption on the PbS(111) surface leads to a lower adsorption energy in comparison to the (100) surface with only −297.34 kJ mol^−1^, indicating that the bridged adsorption sites are less stable. On both cation terminated CdS facets only bridged adsorption sites were found. The adsorption energies of the CdS systems are smaller with −245.03 kJ mol^−1^ and -196.15 kJ mol^−1^ for the (100) and (111) facets, respectively. To compare the removal of thiol ligands from the PbS and PbS/CdS particles, the adsorption energies calculated above can be used. Since our experimentally synthesized PbS particles are mainly covered by (111) facets and our PbS/CdS particles are exclusively covered by CdS(100) facets, the adsorption energies of the PbS(111) surface must be compared with the CdS(100) surface.

Adsorption at PbS(111) surfaces is by 52.31 kJ mol^−1^ stronger than for the CdS(100) surface, which explains a higher affinity of the thiol ligand to the PbS(111) surface as discussed in the experimental results. These results are supported by the bond enthalpies of PbS and CdS with 346 and 208.4 kJ mol^−1^ found in literature, indicating a higher affinity of S to Pb compared to Cd.^51^ Since the CdS shell of the PbS/CdS QDs is relatively thin, the facets will most likely be strained in comparison to the bulk CdS facets used in the calculation. Nevertheless, the results of the calculation should still be applicable, since Zhao *et al.* were able to measure the reflections of zinc blende CdS for thin shell PbS/CdS QDs with a thickness of 1.3 nm.^31^ Furthermore, the lattice mismatch was reported to be only 2%, allowing the formation of CdS facets also for thin shell PbS/CdS QDs.^38^

The described differences in the network structures heavily influence the optical properties of the synthesized nanomaterials. The observed changes and possible reasons are discussed below.

### Optical properties

**Fig. 5:**
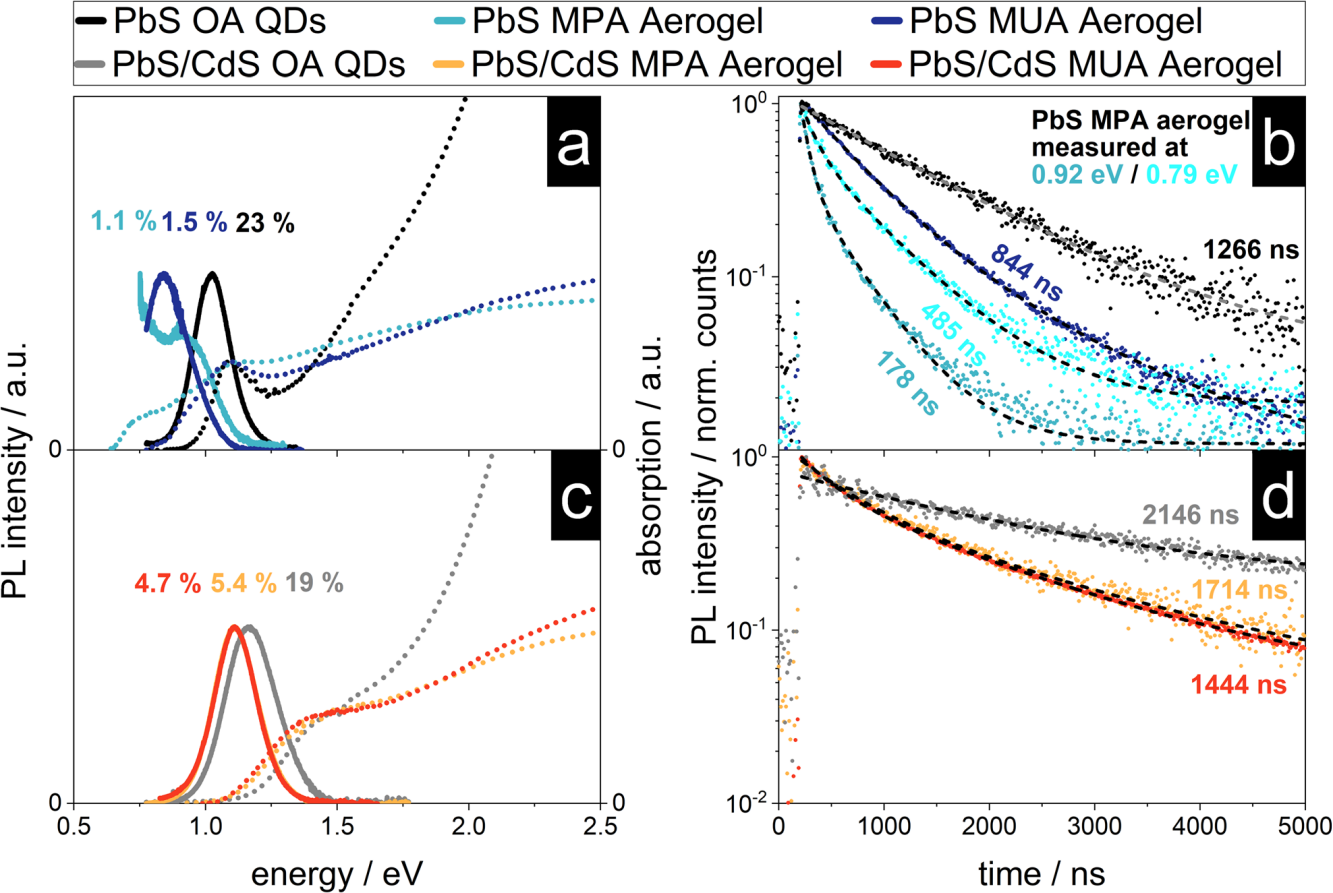
The variety of QDs used during the gelation (PbS or core/shell PbS/CdS) has an impact on the optical properties of the resulting aerogels manifested in bathochromic shifts of the emission and decreased PL lifetimes. Absorption (dotted line) and steady state emission (solid line) spectra and PL decays (dots) (a and c) of PbS OA QDs, PbS MUA aerogel and PbS MPA aerogel and (b and d) core/shell PbS/CdS OA QDs, core/shell PbS/CdS MUA aerogel and core/shell PbS/CdS MPA aerogel. The PLQY and average PL lifetimes *τ̄* are shown. The mono-exponential (for PbS and core/shell PbS/CdS OA QDs) and bi-exponential (for aerogels) fits of the PL decays are displayed as dashed lines.

PbS nanocrystals are known to interact with each other, even in solution and without crystal contacts, which is manifested in their optical properties like the stokes shift.^52^ Previous reports of characterized NC aerogel systems and simple QD films can act as references for the following characterization like the influence of the network formation onto the PL lifetime and observed shifts of the emission signal.^15,17,22,53–56^ In Fig. 5 the absorption and emission spectra as well as the PL decay of the four synthesized aerogels together with the spectra and decays of the as-synthesized QDs (used for the aerogel synthesis) can be found. As described above the steady-state optical properties of the PbS and core/shell PbS/CdS QDs are relatively untouched by the ligand exchange procedure, except for their PLQY. This is dramatically different to the PbS aerogels. The PbS MUA aerogel emission has a bathochromic shift from 1215 nm (1.020 eV) to 1476 nm (0.890 eV) of 261 nm compared to the PbS OA QDs. A similar shift of the absorption was also measured and the calculated bandgap is decreasing from 0.911 eV to 0.729 eV. A bathochromic shift can also be observed for the optical properties of the PbS MPA aerogel. An emission peak at 1378 nm (0.900 eV) can be measured, but in addition after the local maximum the PL intensity increases further until the edge of the spectral range of the detector is reached at 0.75 eV. To understand the reasons for the bathochromic shift of the PbS QD based aerogels, two phenomena have to be distinguished as discussed in the following two paragraphs.

It is known from literature, that PbS QDs are prone to energy transfer, especially in assemblies like closed-packed QD films. When excited charge carriers are able to transfer between particles of the same population, they accumulate in the bigger particles with smaller bandgaps, because there their energy is minimized. This also leads to a bathochromic shift of the emission, since radiative recombination is consequently happening mostly in these bigger particles.^55,56^ Previously reported shifts for close-packed PbS QDs, that were traced back to energy transfer, were roughly around 40 nm.^54^ Hence, this mechanism cannot solely explain the observed shifts for the PbS aerogels in the present work.

Another reason for a bathochromic shift can be the decrease of the bandgap, which would lead to a bathochromic shift of the emission and absorption to lower energies (while in contrast energy transfer would only lead to a shift of the emission). The absorption of the PbS aerogels is shifted to lower energies and in the case of the PbS MPA aerogel two distinct absorption shoulders can be seen. The bandgap is shifted to 0.628 eV, as calculated from the absorption measurement, corresponding to a decrease of 0.480 eV (or 43% in comparison to the PbS MPA QDs). This change in the bandgap is most likely due to the coalescence of particles during gelation and therefore the formation of enlarged crystalline structures with less confined charge carriers. This is supported by the excitation measurements recorded at 0.79 and 0.93 eV, which can be found in Fig. S9 in the ESI.† In addition to the coalescence, energy transfer might also play a role in the shift of the emission, but this has to be investigated further with more advanced spectroscopic techniques in the future, *e.g.* by low temperature steady-state and time-resolved photoluminescence measurements.^62^

In comparison to the optical properties of the PbS aerogels, for the core/shell PbS/CdS aerogels the changes from OA QDs to aerogels are less pronounced. The emission of both the core/shell PbS/CdS MUA and MPA aerogels shift from 1087 nm (1.140 eV) to 1118 nm (1.109 eV) for the MUA and MPA aerogel. This corresponds to a shift of 31 nm, which is about an eighth of the measured shift from PbS OA QDs to PbS MUA aerogel. The same observation can be made for the absorption and the thereof derived bandgaps, only slightly decreasing from 1.086 eV to 1.004 eV and 1.003 eV for the core/shell PbS/CdS MUA and MPA aerogels, respectively. Therefore, the nanoscopic steady-state optical properties of the core/shell PbS/CdS QDs are retained in the macroscopic core/shell PbS/CdS aerogels, which is quite remarkable considering the observations made in the TEM images. It seems like the attachment of the core/shell PbS/CdS QDs to each other and even the coalescence of QDs, which is especially pronounced for the core/shell PbS/CdS MPA aerogel does not dramatically influence the optical properties. Since this is not the case for the PbS aerogels, the reason for the superior retention of optical properties is attributed to the CdS shell. While the contact of the QDs in case of the PbS samples leads to increased delocalization and interparticle interactions, the CdS shell prevents this, acting as a physical and electronical separator of the PbS cores, which are responsible for the optical properties in the NIR range. This is only possible, because the electronic structure of the core/shell PbS/CdS QDs leads to the localization of the excited electron and hole to the PbS cores, which is typical for type I core/shell structures.^1^ As long as the coalescence during the gelation process only influences the shape and thickness of the CdS shell and the PbS cores remain virtually untouched, so do the optical properties. As the optical properties indeed do not change drastically as for core only PbS aerogels, the cores have to be still isolated by fused shells in the coalesced network. In addition, the PLQY of the core/shell PbS/CdS aerogels is higher at around 5% in comparison to the measured 1–1.5% for the PbS aerogels, which can also be attributed to the physical separation of the PbS cores from the surrounding medium by the CdS shell.^1^ Nevertheless, the bathochromic shift of 31 nm (31 meV) of the emission is observed and an explanation might be energy transfer between neighboring QDs. The extent of the observed shift is comparable to the observations made in literature for bathochromic shifts in PbS and core/shell PbS/CdS QD films around 40 and 50 nm.^53,54^ Due to the close proximity of the particles in the aerogel assemblies energy transfer mechanisms such as charge tunneling have to be considered as well.^53^ At the same time the observed PL shift is accompanied by a similar bathochromic shift of the absorption features and a decrease of the underlying bandgap of about 100 meV, suggesting that energy transfer is not responsible. Nevertheless, the proximity of the QDs in the network make the exchange of charge carriers very likely and energy transfer cannot be ruled out fully.

The time-resolved PL measurements for the PbS and core/shell PbS/CdS samples are shown in Fig. 5c and d. The core/shell PbS/CdS MUA and MPA aerogels show a similar average PL lifetime of *τ̄* = 1714 ns and *τ̄* = 1444 ns respectively. The charge carrier lifetimes are shorter in comparison to the OA stabilized colloids, which was previously observed by Rusch *et al.* for ZnSe/ZnS aerogels.^22^ In structures with quasi type II band alignment, such as CdSe/CdS heterostructures, the PL lifetime increases due to the formation of an interconnected particle network and increased delocalization of excited electrons within the network, while the holes are localized in the CdSe cores.^15,57^ The observation for ZnSe/ZnS aerogels with a type I band alignment was that the PL lifetimes decrease in comparison to the colloids. The same behavior of decreasing PL lifetimes with network formation can be seen for the PbS aerogels. A similar observation has been made by Sayevich *et al.* with CdSe aerogels, where the assembly of the NCs lead to faster decay rates.^17^ This decrease was attributed to increased non-radiative deactivation though energy transfer and recombination on surface states. Similar observations were also made in literature, where the increase of the non-radiative decay rate by introduction of additional defect states lead to decreasing PL lifetimes.^58,59^ This is also relevant here, since the PLQY decreases from OA stabilized QDs to aerogels, and therefore non-radiative recombination is increased. Ushakova *et al.* reported, that the PL lifetime of PbS QDs decreases with decreasing bandgap energy.^60^ The size-dependent changes of the PL lifetime were attributed to the energy of a mid-gap state in the QDs, whose energy is size-dependent. The measured decrease of the bandgap for the PbS MUA aerogel from 0.911 eV to 0.729 eV and the average PL lifetime of *τ̄* = 844 ns is comparable to the correlation found in literature. At the same time for the PbS MPA aerogel the opposite effect can be observed, since the average PL lifetime measured at 0.79 eV is longer than the one measured at 0.92 eV for the same sample (485 and 178 ns, respectively). This indicates that the network formation, close-packing and coalescence of the PbS QDs influence the electronic structure of the material which is manifested in observed differences of the optical properties. Finally, energy transfer has to be considered, since it was shown, that the PL lifetimes in close-packed films is shorter compared to colloidal samples.^56^ Furthermore, the observation made for the PbS MPA aerogel, of decreasing PL lifetimes with increasing bandgap is matching the expected behavior in close-packed assemblies. The small particles of the QD population act as donors exhibiting shorter PL lifetimes, while the larger QDs act as acceptors with longer PL lifetimes during energy exchange.^55,61,62^

It was shown before, that Y^3+^-ions remain in the network after gelation and consequent washing steps.^33,63^ Therefore, the influence of these ions onto the optoelectronic properties of the assemblies have to be considered. At this point the precise optoelectronic interactions of the ions with the nanocrystals have not been investigated, because the introduction of the ions always leads to network formation. This makes the separation of the changes induced by network formation and ions very hard. Nevertheless, in a previous study we were able to show, that the PLQY of in this case CdSe/CdS nanorods did not decrease from aqueous colloid to hydrogel when using Y^3+^- or Yb^3+^-ions as gelation agents.^63^ This is in line with the observations made for our systems, since the PLQY from aqueous colloids to the final networks does indeed also increase (as shown in Table S1 in the ESI†), showing that the Y^3+^-ions do not quench the PL significantly.

In conclusion, the effects of network formation and coalescence of particles influence the optical properties of the synthesized materials. The resulting bathochromic shifts from particle coalescence of 260 nm and more observed for the PbS aerogels can be successfully prevented by using core/shell PbS/CdS QDs as building blocks. In addition, the type I band alignment protects the PbS cores electronically, which leads to higher observed PLQY. Nevertheless, further optimization is necessary to reach similar PLQY for the aerogels as for the QD building blocks. The surface ligands directly influence the obtained network structure, which is more pronounced for the PbS aerogels. This is most likely due to the increased binding energy of the ligands to the PbS surface compared to the CdS surface. Coalescence for core/shell PbS/CdS samples can be observed for both MUA and MPA samples since ligand removal is thermodynamically less obstructed. At the same time, the removal of ligands from the PbS surfaces is less likely and coalescence more likely occurs when the distance between the particles is smaller, due to the shorter MPA ligands. In depth analysis of the optical properties are necessary to completely reveal the underlying processes that lead the differences in PL lifetimes and shift of the emission for the PbS and core/shell PbS/CdS aerogels alike. Like mentioned before, low temperature PL measurements as well as more detailed studies of the PL decays are relevant in this regard.

## Conclusions

The synthesis of novel infrared-emitting nanocrystal-based aerogels has been demonstrated. To the best of our knowledge, this is the first time these kind of material is characterized to this extent. A recently published gelation method, using trivalent ions and mercaptocarboxylic acid stabilized NCs was optimized for NIR-emitting PbS and core/shell PbS/CdS QDs. The network structures of the aerogels are significantly influenced by the surface ligands present on the nanoparticles during the gelation and the particle surfaces. The importance of the surface ligands was shown, whereby the shorter 3-mercaptopropionic acid (MPA) ligands lead to more pronounced coalescence of the particles in comparison to the longer 11-mercaptoundecanoic acid (MUA) ligands. The optical characterization of the three-dimensional networks shows retained NIR PL and absorption from their colloidal QD precursors. The PL and absorption of the PbS aerogels are significantly shifted to lower energies and the PL lifetimes are significantly reduced. Instead, for the core/shell PbS/CdS aerogels only small bathochromic shifts of PL and absorption can be observed and PL lifetimes are decreased less severely compared to the PbS aerogels. The increased retention of the optical properties for the PbS/CdS aerogels can be attributed to the physical and electronic protection of the PbS cores by the CdS shell.

## Author contributions

Sample preparation was performed by D. P. The TEM images were recorded by R. T. G., C. W. and P. R. The SEM images and optical characterization was carried out by D. P. The theoretical calculations were performed and the regarding paragraph written by H. K., supervised by J. A. B. The project was initiated and supervised by N. C. B. The original draft was written by D. P. and reviewed and edited by all authors.

## Conflicts of interest

There are no conflicts to declare.

## Supplementary Material

NA-005-D3NA00404J-s001
