# Supplementary material for: Optical properties of NIR photoluminescent PbS nanocrystal-based three-dimensional networks
Source: Nanoscale Adv. 2023 Aug 14;5(18):5005–14. doi: 10.1039/d3na00404j (PMC10496766; doi:10.1039/d3na00404j)
Supplement: NA-005-D3NA00404J-s001 [file NA-005-D3NA00404J-s001.pdf]

Electronic Supplementary Material (ESI) for Nanoscale Advances.
This journal is © The Royal Society of Chemistry 2023

Electronic Supplementary Information

Optical properties of NIR photoluminescent PbS nanocrystal-based three-dimensional networks

*Denis Pluta, Henning Kuper, Rebecca T. Graf, Christoph Wesemann, Pascal Rusch, Joerg August Becker and Nadja C. Bigall**

Experimental Section

Reagents and Materials:

Lead oxide (PbO, 99.99 %) and cadmium oxide (CdO, 99.998 %) were purchased from alfa aesar. Oleic acid (OA, 90 %) was purchased from ABCR. Toluene (puris p.a.) was purchased from Merck. Octadecene (ODE, 90 %), hexamethyldisilane ($(TMS)_2S$, 98 %), methanol (MeOH, 99.8 %), hexane (p.a), tetrachloroethylen (TCE, >99.9 %), diphenylether (DPE, 99 %), 11-mercaptoundeconoic acid (MUA, 95 %), 3-mercaptopropionic acid (MPA, 99 %), acetone (99.8 %), potassium hydroxide (KOH, 85 %), chloroform (99 %), yttrium chloride hexahydrate ($YCl_3 \cdot 6\ H_2O$, 99.99%) and ytterbium chloride hexahydrate ($YbCl_3$ · 6 H2O, 99.9%) were purchased from Sigma-Aldrich. Nitric acid (HNO_3, >69 %) and hydrochloric acid (HCl, ≥37 %) were purchased from Honeywell.

All chemicals were used without further purification.

Synthesis of PbS QDs:

The PbS QDs have been synthesized following a procedure from Zhang *et al.*,[1] which is a modified method from Hines and Scholes.[2] For a typical synthesis, 1.35 g of PbO (6.1 mmol), 27 mL oleic acid (85.5 mmol) and 43.1 mL of ODE (134.7 mmol) were loaded in a 100 mL flask and degassed under vacuum for 30 min at 110 °C with a Schlenk line. After filling the flask with nitrogen and reaching the injection temperature of 95 °C, the heating mantle was removed

and 630 µL $(TMS)_2S$ dispersed in 5 mL ODE were injected rapidly. The reaction mixture was allowed to cool to 30 °C and the PbS QDs were then precipitated with methanol. The particles were redispersed in hexane and precipitated with methanol and centrifuged at 10621 rcf a total of two times and finally dispersed in 7 mL TCE. The lead concentration was determined *via* atomic absorption spectrometry (AAS).

Synthesis of core/shell PbS/CdS QDs:

The core/shell PbS/CdS QDs have been synthesized following a procedure from Zhao *et al.*[3] A 100 mL flask was loaded with 2.06 g CdO (16 mmol), 12.3 mL oleic acid (38.8 mmol) and 32.8 mL DPE (206.1 mmol) and heated to 255 °C under nitrogen. Afterwards the solution was allowed to cool to room temperature and degassed for 10 min at 100 °C under vacuum. After reopening the flask to nitrogen, 50 mL of toluene was added and the solution was heated back to the injection temperature of 100 °C. 0.6 mmol (lead concentration determined *via* AAS) of the previously synthesized PbS QDs were precipitated with roughly 10 mL methanol and redispersed in 3 mL toluene and rapidly injected into the flask. After 30 min of reaction time at 100 °C the reaction solution was allowed to cool to room temperature and precipitated with methanol. After washing twice with hexane and methanol the core/shell PbS/CdS QDs were finally dispersed in 4 mL TCE. The lead concentration was determined *via* AAS.

Ligand exchange of PbS QDs:

The ligand exchange was carried out following a modified procedure from Bagaria et al.[4], which was adapted and optimized by Kodanek *et al.*[5] For the ligand exchange to MUA or MPA 0.7 mL of PbS QDs with a lead concentration of 59.3 g L^{-1} were precipitated with methanol and redispersed in 1.25 mL of hexane. 3.09 mL of a methanolic transfer solution containing 83 mg KOH and 252 mg MUA or 100 µL MPA was added and the mixture was shaken for 2 hours on

an orbital shaker. After centrifugation the PbS QDs were redispersed in 1.25 mL 0.01 M aqueous KOH and 500 µL of chloroform was added. The two-phase system was centrifuged and the upper brown aqueous phase was separated. Methanol was added to precipitated the PbS QDs, and they were finally redispersed in 4 mL 0.01 M aqueous KOH. The lead concertation was determined *via* AAS.

Ligand exchange of core/shell PbS/CdS QDs:

The ligand exchange for core/shell PbS/CdS with MUA and MPA was carried out similarly as described above for the PbS QDs. 1.5 mL of core/shell PbS/CdS QDs with a lead concentration of 12.45 g L^{-1} were precipitated with methanol and redispersed in 560 µL of hexane. 1.39 mL of a methanolic transfer solution containing 29 mg of KOH, and 90 mg MUA or 33.7 µL MPA was added and the solution was shaken for 2 h on an orbital shaker. After centrifugation the core/shell PbS/CdS QDs were redispersed in 560 µL 0.01 M aqueous KOH and 500 µL of chloroform was added. The two-phase system was centrifuged and the upper brown aqueous phase was separated. Methanol was added to precipitate the core/shell PbS/CdS QDs and were finally redispersed in 1.8 mL 0.01 M aqueous KOH. The lead concentration was determined *via* AAS.

Preparation of hydrogels:

The controlled destabilization of the aqueous PbS and core/shell PbS/CdS QDs for the assembly into hydrogels was carried out following an optimized procedure from Zámbó *et al.*[6] To 800 µL of the QD colloid with a lead concentration of 6.636 g L^{-1} in an 2 mL centrifuge tube 100 µL of a 75 mM aqueous $YCl_3 \cdot 6\ H_2O$ solution was added and the mixture was quickly homogenized with a vortex. The samples were stored at room temperature. After 40 h complete gelation was observed for the PbS MUA and MPA QDs, while for the core/shell

PbS/CdS MUA and MPA QDs complete gelation was only reached after the addition of additional 50 µL of 75 mM aqueous $YCl_3 \cdot 6\ H_2O$ after 16 h and another 24 h of gelation time. Subsequently the hydrogels were washed in multiple steps by exchanging the supernatant. After the gelation was complete, the supernatant was exchanged 5 times with distilled water on two days in a row for a total of 10 water washing steps.

Preparation of aerogels:

The solvent of the hydrogels was carefully changed to acetone by exchanging the supernatant with water-acetone mixtures (7:1, 4:1, 1:1 & 1:4 water to acetone volume ratio). The solvogels were washed twice per day with each water-acetone mixture and finally washed a total of 10 times within 5 days with anhydrous acetone as a preparation for supercritical drying. The prepared solvogels in anhydrous acetone were dried in a critical point dryer (Quorum Technologies, E3100). The samples in Eppendorf tubes were transferred to the sample boat of the dryer and the apparatus was sealed. The acetone was replaced by liquid CO_2 by flushing the chamber and the acetone was drained. After flushing the chamber for several minutes the samples were stored in liquid CO_2 overnight. The next day the chamber was again flushed with fresh liquid CO_2. After sealing the chamber, the temperature was raised to 36 °C and the CO_2 was brought to supercritical conditions (supercritical point: 31.1 °C and 73.9 bar). The pressure was reduced carefully until ambient pressure was reached and the apparatus was allowed to cool down to room temperature. Finally, the aerogels were removed from the chamber and stored in a nitrogen filled glovebox.

Atomic absorption spectrometry (AAS):

The lead concentration of the colloidal QDs were determined *via* AAS. 2 mL of concentrated hydrochlorid acid and 6 mL of nitric acid were combined in a glass vial and allowed to form aqua regia. A set of 6 calibration samples were prepared between 0 and 20 mg L^{-1} by adding the appropriate amount of a 2 g L^{-1} lead standard as well as 1 mL of aqua regia to a 50 mL volumetric flask and is made up to 50 mL with distilled water. A volume of 10 µL of colloidal PbS and 70 µL of colloidal core/shell PbS/CdS QDs were transferred to a glass vial, heated to evaporate any solvent and dissolved by adding 1 mL of aqua regia. The dissolved sample was transferred into a 50 mL volumetric flask and made up to 50 mL with distilled water. The measured extinction values of the calibration samples were plotted against their concentration and fitted with a linear function. The linear equation parameters of the fit were used to calculate the lead concentration of the QD samples.

All samples were measured with a AA140 flame atomic absorption spectrometer from Varian operated with an acetylene air mixture at a ratio of 3:7 and a wavelength of 217 nm.

Structural characterization:

The transmission electron microscopy (TEM) and scanning electron microscopy (SEM) images were taken using a FEI Tecnai G2 F20 TMP transmission electron microscope operated at 200 kV and a JEOL JSM-6700F scanning electron microscope operated at 2 kV respectively. TEM samples were prepared on Quantifoil copper grids with carbon films by drop-casting colloidal QD solutions or aerogel dispersions in acetone onto them. The prepared copper grids were cleaned by placing them on an active charcoal and adding ethanol, as described by Li et al.[7] The aerogels were prepared for SEM measurements by deposition of a small amount of sample on 6 mm PELCO Tabs adhesive carbon films from PLANO.

Optical characterization:

For the optical characterization, colloidal QDs in 0.01 M aqueous KOH or TCE were filled in 3 mL quartz cuvettes (10 mm path length) and aerogels were placed in Teflon holders. Emission, excitation and PL decays were measured with an Edinburgh FLS 1000 equipped with a liquid N_2 cooled NIR-PMT InGaAs detector. For the PL decays a EPL 450 pulsed laser (445.1 nm) was used for excitation in multi-channel scaling (MCS) mode with a repetition rate of 50 kHz. The PLQY were measured with the same instrument in an integrating sphere from Edinburgh. The absorbance of the colloidal QDs and absorption of the aerogels were measured with a Cary 5000 UV-Vis-NIR from Agilent Technologies. For these measurements the aerogels were dispersed in $BaSO_4$ and inserted into the integrating sphere.

The PL decays were fitted with mono- (1) or biexponential (2) functions.

$$I(t) = A_1 \cdot e^{-\frac{t}{\tau_1}} \quad (1)$$

$$I(t) = A_1 \cdot e^{-\frac{t}{\tau_1}} + A_2 \cdot e^{-\frac{t}{\tau_2}} \quad (2)$$

For the biexponential decays the fit parameters were used to calculate the average PL lifetime $\bar{\tau}$ using equation (3).

$$\bar{\tau} = \frac{A_1 {\tau_1}^2 + A_2 {\tau_2}^2}{A_1 \tau_1 + A_2 \tau_2} \quad (3)$$

Table S1 Optical property data of the synthesized QDs and aerogels. The FLT decays of the oleate QDs were fitted monoexponentially, the FLT decays of the aerogels were fitted biexponentially. The average lifetimes $\bar{\tau}$ were calculated with the fit data.

Sample	E_{Em} [eV] (λ_{Em} [nm])	Bandgap [eV]	PLQY	$\bar{\tau}$ [ns]
PbS oleate QDs	1.020 (1215)	0.911	23 %	1266
PbS MUA QDs	1.035 (1198)	0.955	[b]	
PbS MPA QDs	*No measurable emission*	0.943	[b]	
PbS/CdS oleate QDs	1.140 (1087)	1.086	19 %	2146
PbS/CdS MUA QDs	1.175 (1055)	1.130	[b]	
PbS/CdS MPA QDs	1.169 (1061)	1.108	[b]	
PbS MUA aerogel	0.840 (1476)	0.729	1.5 %[a]	844
PbS MPA aerogel	0.900 (1378)[c]	0.628	1.1 %[a]	485 @0.79 eV<br>178 @ 0.92 eV
PbS/CdS MUA aerogel	1.109 (1118)	1.004	4.7 %	1714
PbS/CdS MPA aerogel	1.109 (1118)	1.003	5.4 %	1444

[a] Emission signal cut off, estimation is low.; [b] not measureable; [c] highest energy maximum

Adsorption energy calculations:

For both PbS and CdS, surface structures with (100) and (111) facets were generated by slab structures and a model thiol molecule was used. To create a deprotonated thiol ligand model molecule and also to obtain a charge neutral simulation cell, the absorbed molecule consists of a deprotonated thiol group, i.e., a sulfur atom with a n-hexylchaine and trimethyl-amine group at the other end ($S-(CH_2)_6-N-(CH_3)_3$). This model molecule is based on commercially available zwitterionic detergents used, for example, for biospecific and bioselective chromatography.[8] Here it serves as a test molecule to probe the surfaces.

The molecule was placed at a distance of about 5 Å between the Pb/Cd-terminated surface to the sulfur atom of the ligand, with the rest of the ligand oriented orthogonal away from the surface. Through geometry optimization, the thiol ligand moves to a self-selected adsorption site. For the geometry optimization, a 5x5x3 Monkhorst-Pack k-grid mesh is used, and the atomic positions are relaxed until the energy gradient is below 0.001 Hartree/Å.

On the PbS(100) surface, the sulfur atom of the thiol ligand is located on top of a lead atom with a distance of 2.96 Å which matches very well with the bulk distance of Pb-S in the rock salt structure of 3.00 Å. In all three other structures, the sulfur atoms are located between two Pb/Cd atoms forming a bridged adsorption site. Distances of the sulfur atoms to its closest Pb/Cd atoms of the surface are given in Table S2.

Table S2 Adsorption energies, distances between the sulfur atom of the thiol ligand and the closest Pb/Cd atom of the surface structure and the type of adsorption site are shown for the four surface types illustrated in Fig. 4.

	PbS(100)	PbS(111)	CdS(100)	CdS(111)
ΔE_{ads}/kJ/mol	-339.04	-297.34	-245.03	-196.15
d(S-Cd/Pb) / Å	2.96	3.32	2.76	2.68
Type of adsorption site	top	bridge	bridge	bridge

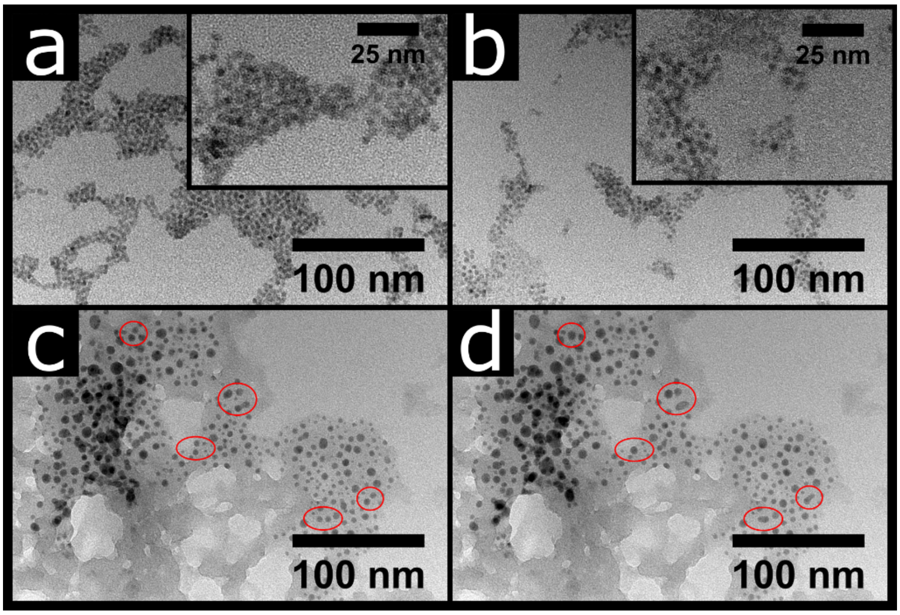

Fig. S1 TEM images of PbS QDs with different ligands. a) PbS OA QDs. b) PbS MUA QDs. c,d) PbS MPA QDs with highlighted sample areas, that coalesce during the measurement (TEM images were taken within 1 min).

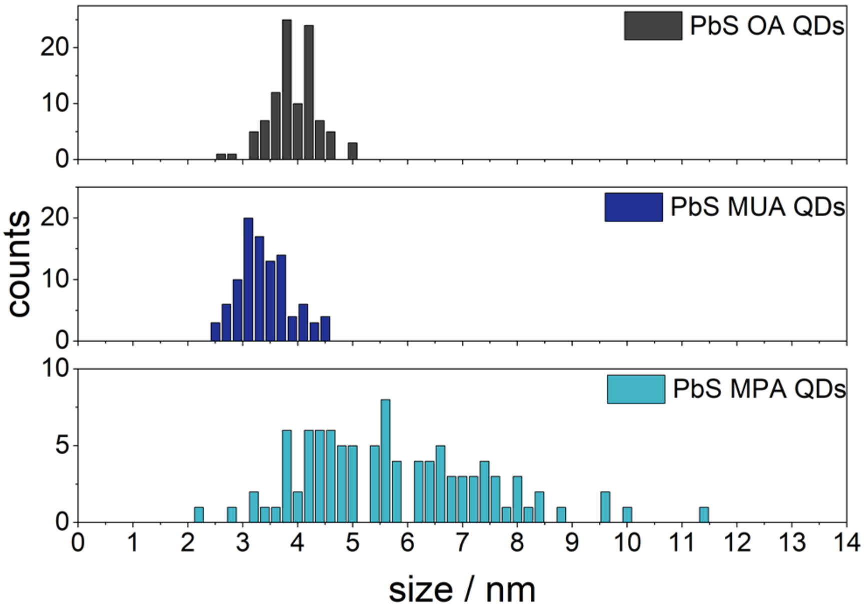

Fig. S2 Size distribution of PbS QDs as synthesized with OA (top) ligands and after ligand exchange with MUA (middle) and MPA (bottom). For each size distribution 100 randomly selected QDs were measured on TEM images.

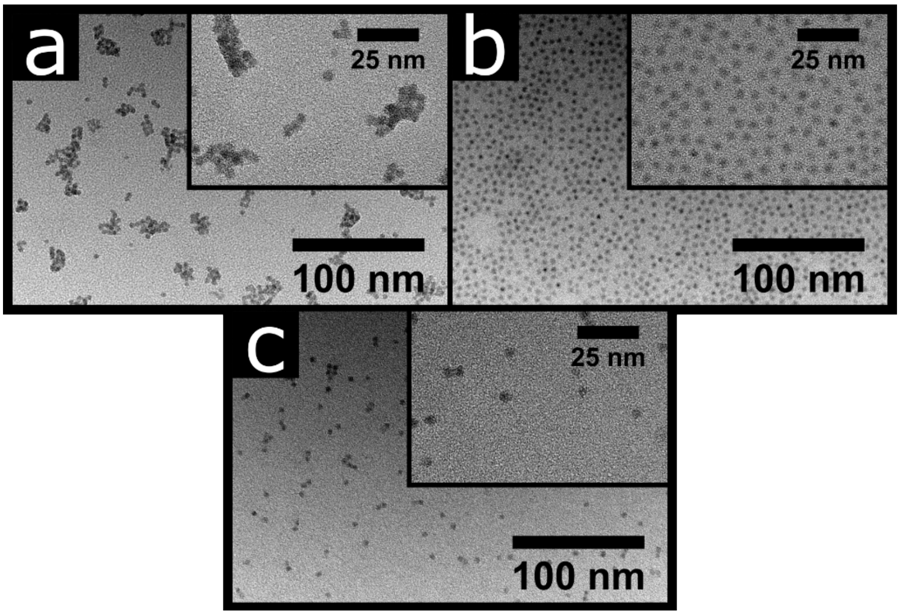

Fig. S3 TEM images of core/shell PbS/CdS QDs with different ligands. a) core/shell PbS/CdS OA QDs. b) core/shell PbS/CdS MUA QDs. c) core/shell PbS/CdS MPA QDs.

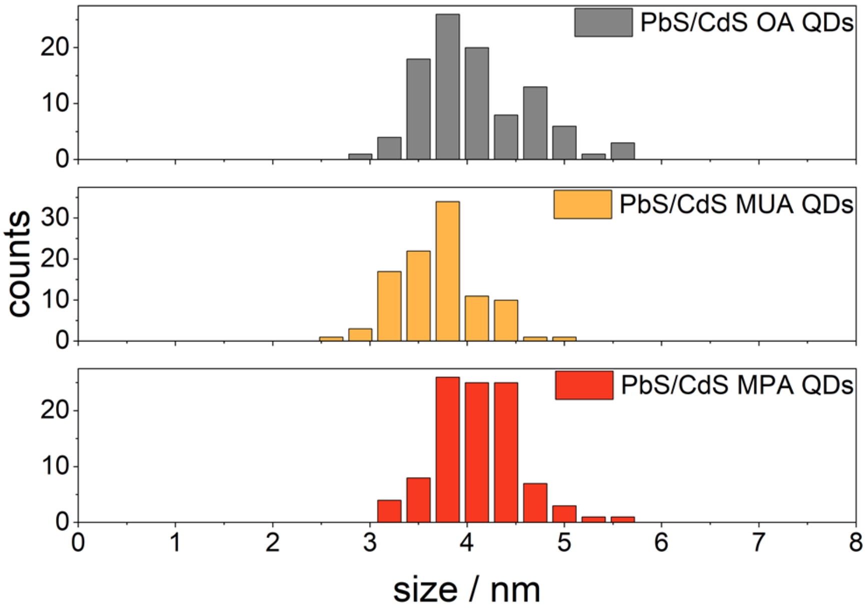

Fig. S4 Size distribution of core/shell PbS/CdS QDs as synthesized with OA (top) ligands and after ligand exchange with MUA (middle) and MPA (bottom). For each size distribution 100 randomly selected QDs were measured on TEM images.

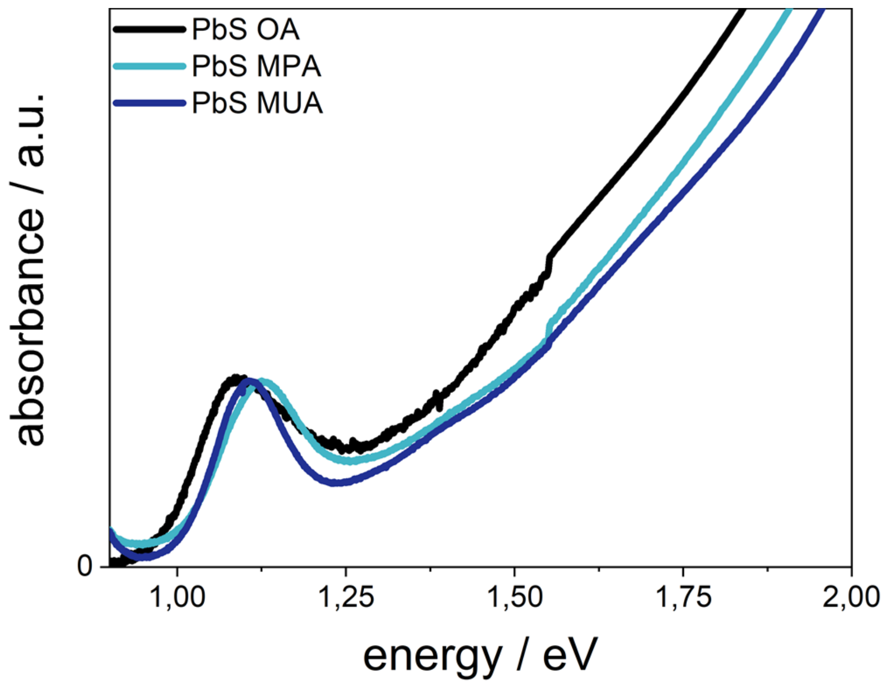

Fig. S5 Normalized absorbance spectra of PbS QDs, stabilized with OA, MPA and MUA ligands. The PbS OA sample is measured in TCE, the PbS MUA and PbS MPA samples are measured in 0.01 M aqueous KOH. The increase in absorbance for the aqueous samples below the first excitation maximum is due to the absorption of water.

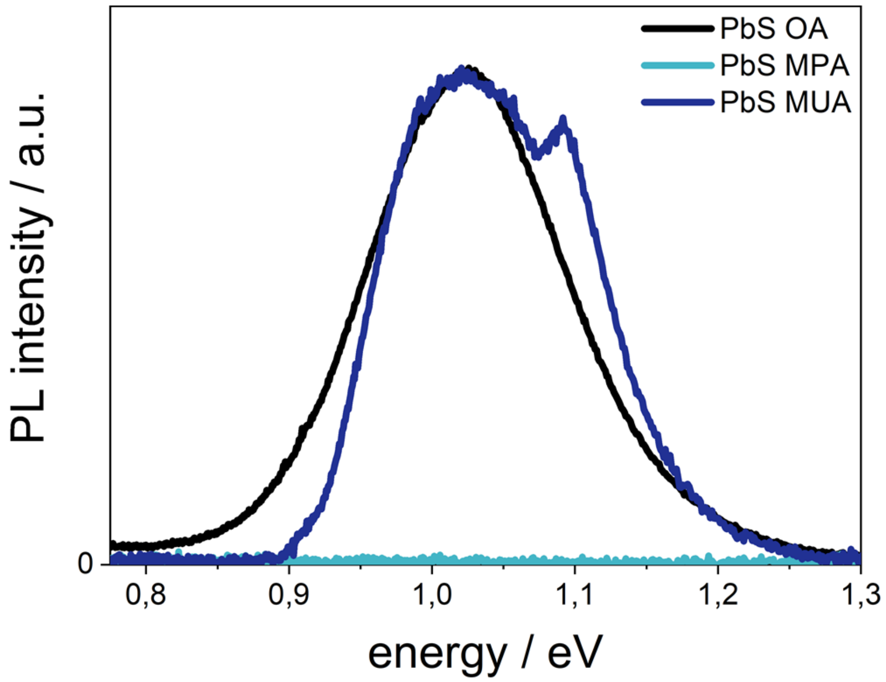

Fig. S6 Normalized emission spectra of PbS QDs, stabilized with OA, MPA and MUA ligands. The PbS OA sample is measured in TCE, the PbS MUA and PbS MPA samples are measured in 0.01 M aqueous KOH. The emission of the aqueous samples is cut of at 0.9 eV due to the absorption of water.

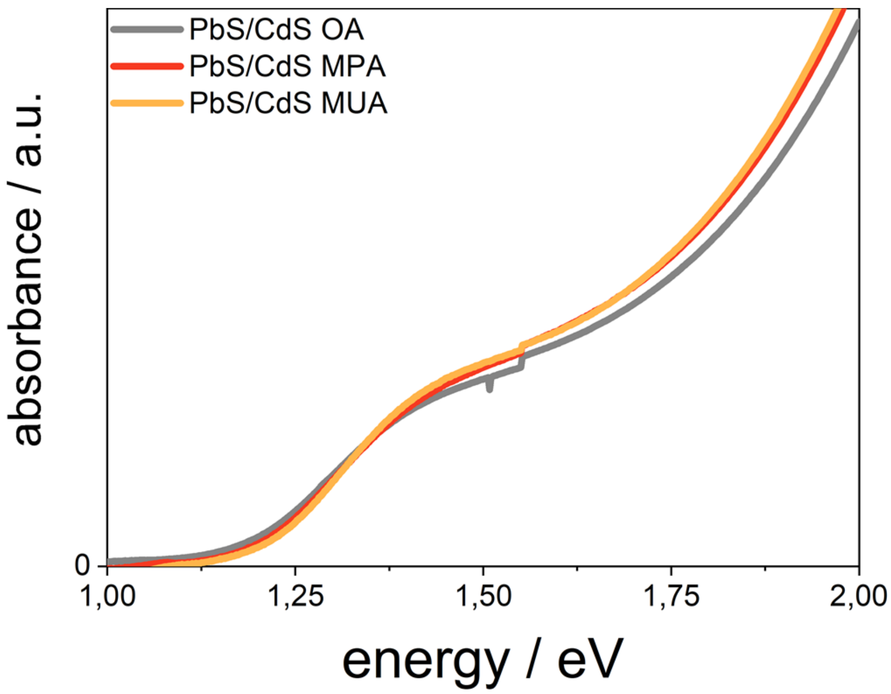

Fig. S7 Absorbance spectra of core/shell PbS/CdS QDs, stabilized with OA, MPA and MUA ligands. The core/shell PbS/CdS OA sample is measured in TCE, the PbS MUA and PbS MPA samples are measured in 0.01 M aqueous KOH.

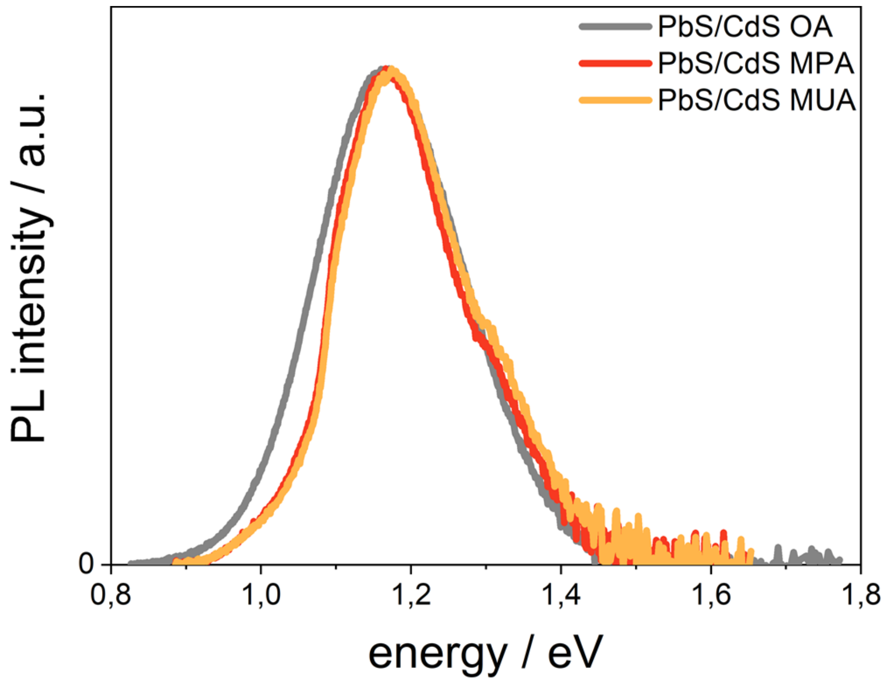

Fig. S8 Normalized emission spectra of core/shell PbS/CdS QDs, stabilized with OA, MPA and MUA ligands. The core/shell PbS/CdS OA sample is measured in TCE, the core/shell PbS/CdS MUA and core/shell PbS/CdS MPA samples are measured in 0.01 M aqueous KOH. The emission of the aqueous samples is influenced by the absorption of water, leading to the asymmetric shape of the emission signal.

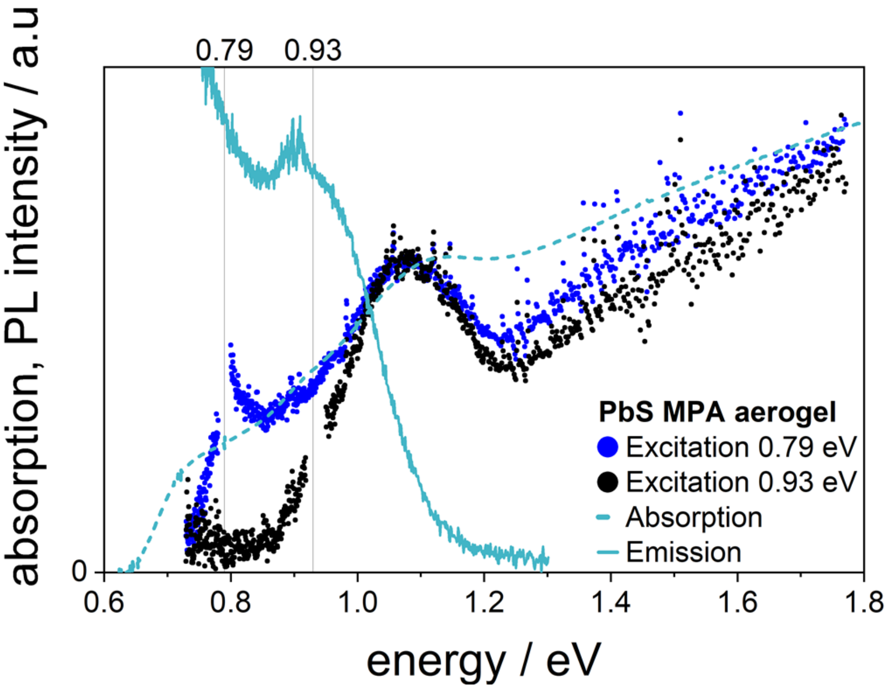

Fig. S9 Excitation spectra of the PbS MPA aerogel, recorded at 0.79 eV (blue) and 0.93 eV (black). The absorption and emission are shown in light blue. Both excitation measurements show a maximum at 1.08 eV, but the measurement recorded at 0.79 eV shows increased counts below the maximum in comparison to the measurement recorded at 0.79 eV.

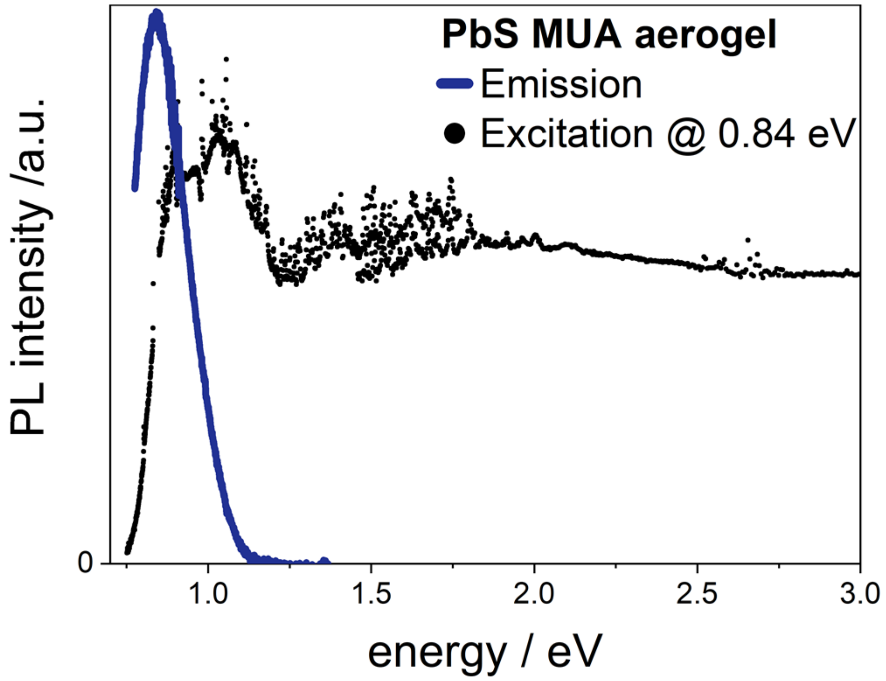

Fig. S10 Excitation spectrum of the PbS MUA aerogel, recorded at 0.84 eV (black) and the emission (dark blue).

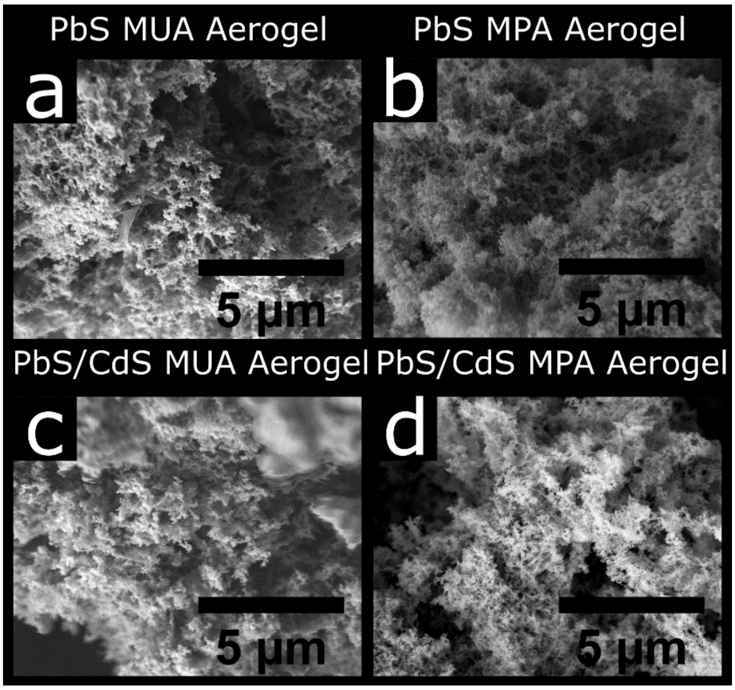

Fig. S11 SEM images of the PbS and core/shell PbS/CdS aerogels synthesized from PbS and core/shell PbS/CdS MUA and MPA QDs.

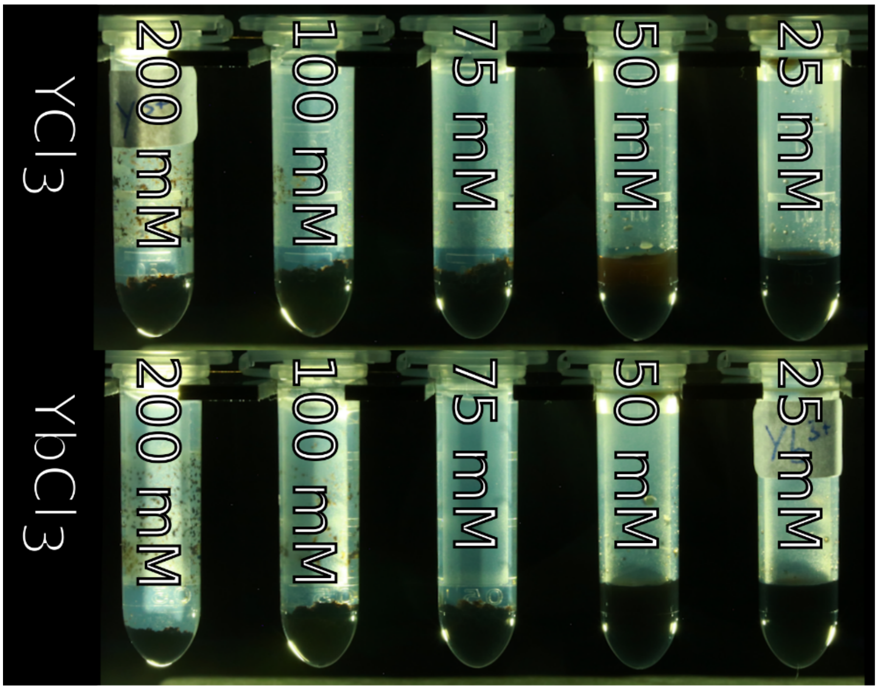

Fig. S12 PbS MUA hydrogels gelled with $YbCl_3$ and YCl_3 with concentrations from 25 to 200 mM. The gels were synthesized with 400 µL of PbS QDs (c_{Pb} = 6.636 mg/mL) and 50 µL of $YbCl_3$/YCl_3 solution.

References

[1] J. Zhang, R. W. Crisp, J. Gao, D. M. Kroupa, M. C. Beard, J. M. Luther, *J. Phys. Chem. Lett.* **2015**, *6*, 1830.

[2] M. A. Hines, G. D. Scholes, *Adv. Mater.* **2003**, *15*, 1844.

[3] H. Zhao, M. Chaker, N. Wu, D. Ma, *J. Mater. Chem.* **2011**, *21*, 8898.

[4] H. G. Bagaria, E. T. Ada, M. Shamsuzzoha, D. E. Nikles, D. T. Johnson, *Langmuir* **2006**, *22*, 7732.

[5] T. Kodanek, H. M. Banbela, S. Naskar, P. Adel, N. C. Bigall, D. Dorfs, *Nanoscale* **2015**, *7*, 19300.

[6] D. Zámbó, A. Schlosser, P. Rusch, F. Lübkemann, J. Koch, H. Pfnür, N. C. Bigall, *Small* **2020**, *16*, 1906934.

[7] C. Li, A. P. Tardajos, D. Wang, D. Choukroun, K. Van Daele, T. Breugelmans, S. Bals, *Ultramicroscopy* **2021**, *221*, 113195.

[8] K. H. Nollstadt, Y. D. Karkhanis, M. J. Gnozzio, M. St.J. Crane, A. M. Gurnett, D. M. Schmatz, M. J. Turner, *J. Chromatogr. B Biomed. Sci. Appl.* **1989**, *497*, 87.
